# Adverse Childhood Experiences and Health at Age 50 Years in the National Child Development Study

**DOI:** 10.1001/jamanetworkopen.2025.25708

**Published:** 2025-08-28

**Authors:** Kate A. Timmins, Ross MacDonald, Marcus Beasley, Gary J. Macfarlane

**Affiliations:** 1 Aberdeen Centre for Arthritis and Musculoskeletal Health (Epidemiology Group), University of Aberdeen, Aberdeen, United Kingdom; 2 Population Health Sciences Institute, Newcastle University, Newcastle upon Tyne, United Kingdom

## Abstract

**Importance:**

Most studies have reported associations between adverse childhood experiences (ACEs) and a single health outcome. It is therefore difficult to assess impacts holistically. Examining a broad range of health outcomes may help inform the targeting of interventions.

**Objective:**

To quantify the modeled association of ACEs with several adult health outcomes within a single study.

**Design, Setting, and Participants:**

This cohort study using an outcome-wide approach was a secondary analysis of the National Child Development Study, a longitudinal birth cohort of people across the UK born during 1 week in 1958. Data from the follow-up survey at age 50 years were used. The data analysis was conducted between September and October 2024.

**Exposures:**

Fourteen ACEs from data collected throughout childhood (age 7, 11, and 16 years) and retrospectively (age 23, 33, and 44 years).

**Main Outcomes and Measures:**

Eleven outcomes (with prevalence ≥5%) self-reported at age 50 years were examined using doubly robust estimation and included severe pain, poor mental health, asthma or bronchitis, hay fever or rhinitis, back problems, hearing problems, eyesight problems, hypertension, migraine, skin problems, and gastrointestinal problems. Risk differences were estimated between pseudopopulations (exposure to ACEs set at 100% vs 0%) with adjustment made for confounders at birth using inverse probability weights.

**Results:**

From a total of 17 638 people initially included in the birth cohort, 53% participated at age 50 years. Data for 16 321 participants (51.0% men) were analyzed. Adverse childhood experiences were associated with several health outcomes, with the largest risk differences (comparing exposed vs unexposed pseudopopulations) observed for severe pain and poor mental health. In men, the risk of severe pain was 8.70% vs 4.88%, respectively (risk difference, 3.82%; 95% CI, 2.23%-5.42%), and of poor mental health, 10.53% vs 6.68%, respectively (risk difference, 3.85%; 95% CI, 2.16%-5.85%). In women, the risks were 11.22% vs 7.53% (risk difference, 3.69%; 95% CI, 1.71%-5.67%) and 19.10% vs 12.59% (risk difference, 6.50%; 95% CI, 4.13%-8.88%), respectively. Experiencing 4 or more ACEs increased these risks. Migraine, hay fever or rhinitis, eyesight problems, and skin problems showed no association with ACEs. Abuse, neglect, and family conflict showed the most wide-ranging associations.

**Conclusions and Relevance:**

In this cohort study of ACEs and health conditions at age 50 years, the findings suggest that while broad-spectrum interventions remain important to ameliorate the influence of ACEs, a targeted approach that considers the types of ACEs may address specific vulnerabilities, particularly poor mental health and severe pain.

## Key Points

**Question:**

What can we tell from the magnitudes of associations between adverse childhood experiences (ACEs) and different health outcomes at age 50 years?

**Findings:**

In this cohort study of 16 321 participants in the National Child Development Study, ACEs showed associations of differing magnitude with individual health outcomes. The size and pattern of excess risk differed between men and women; however, severe pain and poor mental health showed the largest excess risks in both sexes.

**Meaning:**

These findings suggest that ACEs may not have an indiscriminate association with later poor health and that a targeted approach may be warranted to address specific vulnerabilities (eg, severe pain), while broad-spectrum interventions remain important to ameliorate the influence of ACEs on multiple outcomes.

## Teaser

This cohort study examines the association of adverse childhood experiences with several adult health outcomes at age 50 years among UK National Child Development study participants.

## Introduction

Adverse childhood experiences (ACEs) refer to potentially traumatic events or circumstances during childhood.^1^ As well as affecting childhood well-being, ACEs have been associated with long-term adverse health and social outcomes.^2,3^ A meta-analysis of adult health outcomes reported positive associations of having 4 or more ACEs with all 23 outcomes analyzed.^4^

Issues of confounding and recall bias challenge our interpretation of much of the available evidence. A further challenge is the heterogeneity in studies, including varied definitions and assessment of ACEs. There is no consensus about what constitutes an ACE. The Centers for Disease Control and Prevention–Kaiser Permanente ACE study questionnaire^2^ assessed 10 ACEs (abuse, neglect, and 4 other household challenges). Since, researchers have argued for the inclusion of a broader set of experiences,^5^ and evidence suggests that experience of being bullied, poverty, and racism, among others, are associated with outcomes such as chronic pain.^3,6,7^

The focus of the Consortium Against Pain Inequality (CAPE) is understanding the association between ACEs and adult chronic pain. There have been credible hypotheses for biological and/or psychological mechanisms that may link ACEs to the development of chronic pain specifically.^8^ Alternatively, childhood psychosocial stressors may be broad-spectrum risk factors^9^ for a variety of adverse outcomes, of which chronic pain is one. Establishing whether ACEs are broad-spectrum risk factors or whether there are specific experiences that increase the risk of pain would aid in the design and implementation of appropriate interventions. Use of a single prospective cohort allows us to quantify the relative associations between ACEs and several outcomes, but with similar adjustment for confounding, and has been described as an outcome-wide approach.^10^

The aim of this study was to quantify magnitudes of association within the same cohort between reported ACEs and a range of poor health outcomes. Specifically, we wanted to determine what factors are associated with pain and whether specific types of childhood adversity are associated with poor health outcomes.

## Methods

This cohort study examined data from the National Child Development Study (NCDS), also known as the 1958 Birth Cohort Study,^11^ which follows approximately 17 000 people born during 1 week in 1958 in England, Scotland, and Wales. The NCDS collected data perinatally, with current data available from 10 sweeps of follow-up through age 62 years.^12^ This study analyzed data up to when participants were aged 50 years, when questions on the experience of pain were included. We excluded participants who died before age 50 years in the main analyses but considered the association between ACEs and mortality in a sensitivity analysis. Response rates for each wave of the NCDS are published online, as are details of the ethical approval and consent procedures.^13^ As is standard for UK longitudinal cohorts held by the UK Data Service, no additional ethical review was required for approved researchers to perform statistical analyses on anonymized data. We followed the Strengthening the Reporting of Observational Studies in Epidemiology (STROBE) reporting guideline.^14^

At age 50 years, participants were interviewed about their current health, using a list of 16 conditions. We included 9 outcomes for which the prevalence was 5% or more to allow sufficient events for each outcome and to reduce the likelihood of model nonconvergence. These outcomes were asthma or wheezy bronchitis (hereafter, asthma or bronchitis); seasonal or perennial allergic rhinitis (hereafter referred to as hay fever or rhinitis); recurrent backache, prolapsed disc, or sciatica (hereafter referred to as back problems); hearing problems; eyesight problems; hypertension; migraine; eczema or other skin problems (hereafter referred to as skin problems); and gastrointestinal problems (eg, stomach, bowel, gall bladder).

Participants were asked if they had seen a physician or specialist or had been to a hospital about a mental health problem and about how much bodily pain they experienced in the past 4 weeks (to which they could respond none, very mild, mild, moderate, severe, or very severe). We derived a variable for severe pain that included responses of severe and very severe. We originally intended to include pain interference alongside pain severity, but the results were so similar that we only present data on severity.

Data on ACEs were drawn from 3 waves throughout childhood (at ages 7, 11, and 16 years) and retrospectively at ages 23, 33, and 44 years. We identified 14 adverse experiences (eTable 1 in Supplement 1). Individual ACEs were marked as present if any of the component variables were present and as missing only if data were missing for all component variables. The presence of any of the 14 ACEs was our main exposure variable. We additionally used a threshold of 4 or more ACEs to define polyadversity^2,4^ and a variable to indicate the presence of any of the 10 ACEs included in the Centers for Disease Control and Prevention–Kaiser Permanente ACE study questionnaire^2^ (hereafter referred to as the original set of ACEs). These ACEs were physical, emotional, and sexual abuse; physical and emotional neglect; household substance abuse; mental illness; domestic violence; parental separation; and criminal behavior.

As recommended by VanderWeele et al,^10^ we selected covariates that preceded the exposure (ie, measured at birth) rather than using a directed acyclic graph. This approach prevents overadjustment in which confounders measured subsequent to exposure may be mediators, and it allowed us to hold constant the confounders across health outcomes. We removed variables that displayed collinearity to leave 5 potential confounders, which included birthweight, mother’s age at birth, mother’s smoking during pregnancy, mother’s schooling beyond minimum age, and overcrowding in the home. Men and women were analyzed separately. We were unable to stratify by racial and ethnic groups due to the low diversity within the sample.

### Patient and Public Involvement

The CAPE benefits from the input of the Chronic Pain Advisory Group, which comprises people with lived experience of chronic pain and childhood adversity. They received compensation for their time in line with National Institute for Health and Care Research involvement guidelines.^15^

### Statistical Analysis

We adopted a potential outcomes framework^16^ to compare estimated risks of the outcome for counterfactual scenarios to quantify the effect of simulated exposure. The potential outcomes framework estimates the difference between 2 hypothetical pseudopopulations: one in which the exposure is set to 0 and the other in which it is set to 1 for all. We planned our analyses around a prespecified estimand (ie, the mean risk difference of each health outcome in adulthood [age 50 years] for participants reporting ≥1 ACE compared with no ACEs), which defined our targets for estimation and linked them explicitly to our aims.^17^ For brevity, we have not listed separate estimands for each outcome.

We contrasted counterfactual outcomes using doubly robust estimation of risk differences.^18^ We created inverse probability weights,^16^ incorporating the probability of being exposed conditional on the 5 confounders (as estimated by logistic regression models) separately for men and women. We estimated risk differences by comparing outcome models. Poisson models, with inverse probability weights, were applied to pseudopopulations in which the exposure was set to either all unexposed or all exposed. We modeled each outcome separately, holding the exposure and inverse probability weights constant. We repeated the analyses modeling exposure to polyadversity (≥4 ACEs); each of the 14 ACE types individually; and any (≥1 vs none) of the original set of ACEs.

We conducted a separate analysis with death (before age 50 years) as the outcome to gauge the extent of survivor bias. We estimated the mortality risk ratio using Poisson regression with robust standard errors, adjusted for the same set of confounders as the main analyses. We excluded deaths prior to age 7 years, the first age at which an ACE could be recorded. Analyses were conducted from September to October 2024, using Stata, version 15.1 (StataCorp LLC).^19^

eTable 2 in Supplement 1 shows differences between complete and noncomplete cases. We used multiple imputation to address missing data (eMethods in Supplement 1). The imputation model included all outcomes, exposures, baseline confounders, and 7 auxiliary variables (eTable 3 in Supplement 1). We imputed values separately for men and women. Each (derived) ACE was imputed; overall ACE exposure and summary scores were derived following imputation.

## Results

A total of 16 321 people from the NCDS were alive at the 50-year follow-up (49.0% female and 51.0% male; 98.8% of White race, and 1.2% of races and ethnicities other than White). A total of 8516 participants (52.2%) completed the questionnaire about pain, while 9431 (57.8%) took part in the interview about other health conditions. The prevalence of outcomes ranged from 8.2% (skin problems, gastrointestinal problems, and severe pain) to 67.1% (eyesight problems). The most common ACEs reported were neglect (4280 participants [27.2%]), illness of a family member (3971 participants [25.2%]), and financial difficulties (3229 participants [21.7%]). A total of 5981 participants (36.6%) reported at least 1 of the original set of ACEs, 11 039 (67.6%) reported at least 1 ACE in the wider set, and 1855 (11.4%) reported 4 or more ACEs (Table 1).

**Table 1.** Participant Characteristics

Characteristic	Participants, No. (%)			
	Men	Women	Total	Missing^a^
Full sample	8331 (51.0)	7990 (49.0)	16 321 (100)	0
ACEs				
≥1	5639 (67.7)	5400 (67.6)	11 039 (67.6)	0
≥4	869 (10.4)	986 (12.3)	1855 (11.4)	0
≥1 Original set of ACEs	3142 (37.7)	2839 (35.5)	5981 (36.6)	0
Abuse	408 (9.3)	531 (11.8)	939 (10.6)	7454 (45.7)
Neglect	2344 (29.2)	1936 (25.0)	4280 (27.2)	559 (3.4)
Witnessing abuse	187 (4.3)	332 (7.4)	519 (5.9)	7454 (45.7)
Substance abuse (family member)	567 (7.8)	713 (10.1)	1280 (8.9)	2002 (12.3)
Criminal activity (family member)	118 (1.7)	123 (1.8)	241 (1.7)	2504 (15.3)
Mental illness (family member)	462 (5.9)	400 (5.3)	862 (5.6)	822 (5.0)
Illness (family member)	2016 (25.2)	1955 (25.3)	3971 (25.2)	592 (3.6)
Family conflict	814 (10.5)	982 (13.2)	1796 (11.8)	1139 (7.0)
Divorce or separation of parents	1062 (13.2)	1098 (14.2)	2160 (13.7)	554 (3.4)
Separation from parent	400 (5.0)	354 (4.6)	754 (4.8)	592 (3.6)
Death of a parent	1096 (13.7)	1079 (14.0)	2175 (13.8)	592 (3.6)
Being bullied	616 (8.1)	494 (6.7)	1110 (7.4)	1332 (8.2)
Financial difficulties	1677 (22.1)	1552 (21.3)	3229 (21.7)	1464 (9.0)
Being kept off school	445 (8.1)	766 (14.4)	1211 (11.2)	5542 (34.0)
Health outcome				
Severe pain in past 4 weeks	289 (7.1)	409 (9.3)	698 (8.2)	7844 (48.1)
Poor mental health	424 (9.1)	819 (17.1)	1243 (13.1)	6864 (42.1)
Asthma or bronchitis	384 (8.3)	541 (11.3)	925 (9.8)	6894 (42.2)
Hay fever or rhinitis	615 (13.3)	669 (14.0)	1284 (13.6)	6894 (42.2)
Back problems	809 (17.4)	836 (17.5)	1645 (17.4)	6894 (42.2)
Hearing problems	556 (12.0)	361 (7.5)	917 (9.7)	6894 (42.2)
Eyesight problems	3034 (65.4)	3288 (68.7)	6322 (67.1)	6894 (42.2)
Hypertension	776 (16.7)	645 (13.5)	1421 (15.1)	6894 (42.2)
Migraine	229 (4.9)	567 (11.8)	796 (8.4)	6894 (42.2)
Skin problems	352 (7.6)	422 (8.8)	774 (8.2)	6894 (42.2)
Gastrointestinal problems	328 (7.1)	447 (9.3)	775 (8.2)	6894 (42.2)

Abbreviation: ACE, adverse childhood experience.

^a^ Percent missing of all participants alive at age 50 years.

Of the original cohort, 1435 participants (8.1%) died prior to age 50 years. Male sex, low birthweight, younger age of mother at time of childbirth, mother smoking prior to or during pregnancy, and overcrowding in the household at the time of childbirth were all associated with a greater risk of mortality (eTable 4 in Supplement 1). The reporting of any ACE was associated with an increased risk of mortality before age 50 years in adjusted analysis (mortality risk ratio, 1.22; 95% CI, 1.01-1.47) (eTable 4 in Supplement 1).

**Figure 1.**
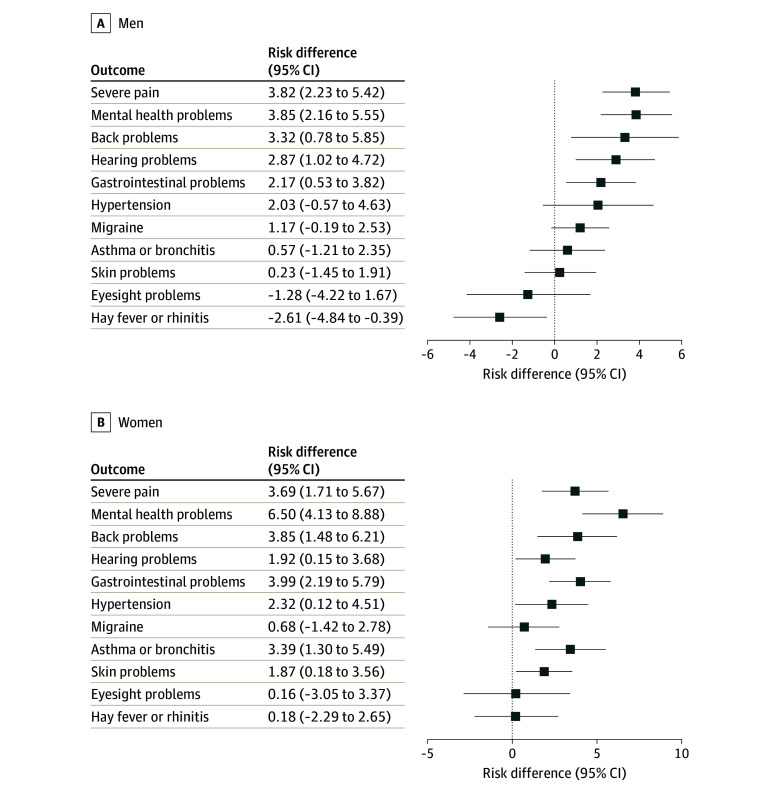
Risk Differences for Individual Health Outcomes, Comparing Pseudopopulations Exposed or Unexposed to at Least 1 Adverse Childhood Experience (ACE)

In men, the excess risk for health conditions associated with reporting at least 1 ACE (comparing exposed vs unexposed pseudopopulations) was greatest for severe pain (8.70% vs 4.88%; risk difference, 3.82%; 95% CI, 2.23%-5.42%), poor mental health (10.53% vs 6.68%; risk difference, 3.85%; 95% CI, 2.16%-5.55%), and back problems (19.11% vs 15.80%; risk difference, 3.32%; 95% CI, 0.78%-5.85%) (Figure 1A; eTable 5 in Supplement 1). In contrast, the excess risk was low for asthma or bronchitis (8.93% vs 8.36%; risk difference, 0.57%; 95% CI, −1.21% to 2.35%) and skin problems (7.88% vs 7.65%; risk difference, 0.23%; 95% CI, −1.45% to 1.91%), and there was no excess risk for eyesight problems and hay fever or rhinitis. In women, the greatest excess risk was for poor mental health (19.10% vs 12.59%; risk difference, 6.50%; 95% CI, 4.13%-8.88%), with excess risk also found for severe pain (11.22% vs 7.53%; risk difference, 3.69%; 95% CI, 1.71%-5.67%), back problems (18.97% vs 15.12%; risk difference, 3.85%; 95% CI, 1.48%-6.21%), gastrointestinal problems (10.63% vs 6.64%; risk difference, 3.99%; 95% CI, 2.19%-5.79%), and asthma or bronchitis (12.60% vs 9.21%; risk difference, 3.39%; 95% CI, 1.30%-5.49%). For migraine, eyesight problems, and hay fever or rhinitis, the risk differences were small and not significant (Figure 1B; eTable 5 in Supplement 1).

Among health outcomes associated with ACEs, reporting polyadversity had the highest excess risk, and the pattern of risk for the original set and wider definition of ACEs was very similar (Table 2). The highest excess risks for 4 or more ACEs in both men and women were associated with poor mental health, with risk differences of 8.36% (95% CI, 4.58%-12.14%) and 11.35% (95% CI, 7.61%-15.08%), respectively, while the excess risk differences associated with severe pain were just slightly lower at 6.32% (95% CI, 3.16%-9.48%) and 7.19% (95% CI, 4.17%-10.21%), respectively. For men, reporting polyadversity was also associated with a risk difference of 5.59% (95% CI, 1.12%-10.06%) for back problems and 5.70% (95% CI, 2.61%-8.80%) for gastrointestinal problems, while for women, polyadversity was associated with a risk difference of 4.59% (95% CI, 1.52%-7.67%) for asthma or bronchitis, 5.02% (95% CI, 2.34%-7.70%) for hearing problems, 4.39% (95% CI, 0.60%-8.18%) for back problems, 8.27% (95% CI, 5.27%-11.27%) for gastrointestinal problems, and 5.66% (95% CI, 2.44%-8.88%) for hypertension. Reporting of polyadversity was not associated with a significant excess risk (in either sex) for hay fever or rhinitis, eyesight problems, skin problems, and migraine.

**Table 2.** Risk Differences According to Number of ACEs Reported

Health outcome	Risk difference, % (95% CI)					
	≥1 ACE		≥1 Original set of ACEs		≥4 ACEs	
	Men	Women	Men	Women	Men	Women
Severe pain	3.82 (2.23 to 5.42)	3.69 (1.71 to 5.67)	4.77 (2.97 to 6.57)	3.54 (1.63 to 5.45)	6.32 (3.16 to 9.48)	7.19 (4.17 to 10.21)
Poor mental health	3.85 (2.16 to 5.55)	6.50 (4.13 to 8.88)	5.18 (3.40 to 6.96)	6.50 (4.19 to 8.82)	8.36 (4.58 to 12.14)	11.35 (7.61 to 15.08)
Asthma or bronchitis	0.57 (−1.21 to 2.35)	3.39 (1.30 to 5.49)	1.12 (−0.76 to 3.00)	3.72 (1.60 to 5.83)	2.94 (−0.07 to 5.95)	4.59 (1.52 to 7.67)
Hay fever or rhinitis	−2.61 (−4.84 to −0.39)	0.18 (−2.29 to 2.65)	−2.88 (−4.80 to −0.97)	0.27 (−1.88 to 2.42)	−4.29 (−7.35 to −1.22)	2.89 (−0.21 to 5.97)
Back problems	3.32 (0.78 to 5.85)	3.85 (1.48 to 6.21)	3.41 (0.71 to 6.11)	3.84 (1.20 to 6.48)	5.59 (1.12 to 10.06)	4.39 (0.60 to 8.18)
Hearing problems	2.87 (1.02 to 4.72)	1.92 (0.15 to 3.68)	3.57 (1.51 to 5.64)	1.43 (−0.48 to 3.35)	3.70 (0.14 to 7.25)	5.02 (2.34 to 7.70)
Eyesight problems	−1.28 (−4.22 to 1.67)	0.16 (−3.05 to 3.37)	−1.42 (−4.71 to 1.86)	−1.20 (−4.04 to 1.64)	1.25 (−3.51 to 6.02)	−0.74 (−5.27 to 3.79)
Hypertension	2.03 (−0.57 to 4.63)	2.32 (0.12 to 4.51)	2.14 (−0.46 to 4.74)	1.96 (0.02 to 3.90)	2.84 (−1.21 to 6.88)	5.66 (2.44 to 8.88)
Migraine	1.17 (−0.19 to 2.53)	0.68 (−1.42 to 2.78)	0.98 (−0.48 to 2.44)	1.46 (−0.55 to 3.48)	1.38 (−1.45 to 4.20)	1.39 (−1.52 to 4.30)
Skin problems	0.23 (−1.45 to 1.91)	1.87 (0.18 to 3.56)	−2.14 (−3.71 to −0.57)	2.40 (0.63 to 4.18)	−0.98 (−3.73 to 1.77)	1.40 (−1.23 to 4.03)
Gastrointestinal problems	2.17 (0.53 to 3.82)	3.99 (2.19 to 5.79)	1.98 (0.20 to 3.76)	4.84 (3.13 to 6.54)	5.70 (2.61 to 8.80)	8.27 (5.27 to 11.27)

Abbreviation: ACE, adverse childhood experience.

**Figure 2.**
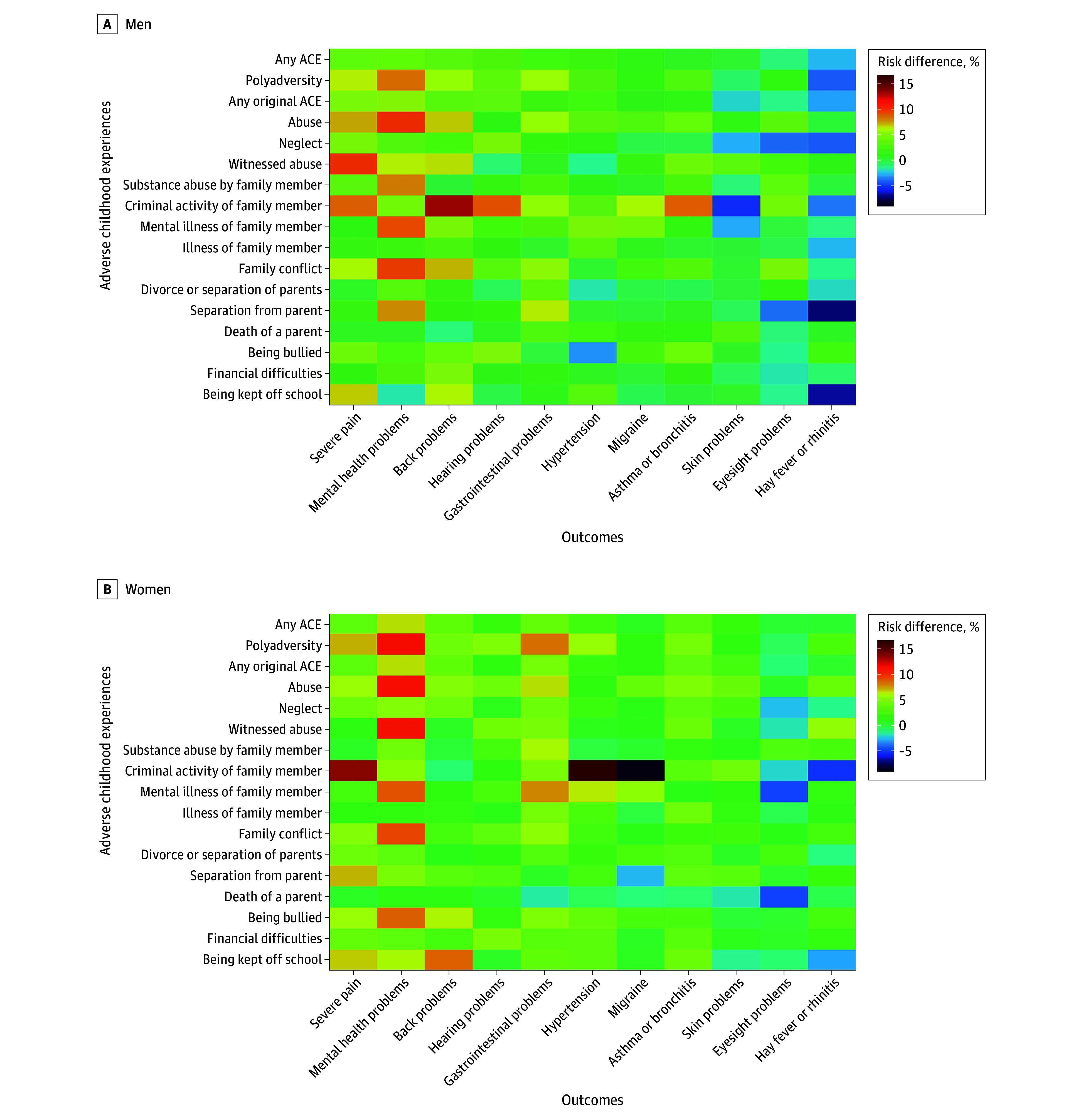
Risk Differences for Individual Health Outcomes, Comparing Pseudopopulations Exposed or Unexposed to Different Categories of Adverse Childhood Experiences (ACEs) Individual ACEs were determined from a mix of prospective and retrospective data. The ACEs of criminal activity of family member, mental illness of family member, illness of family member, death of a parent, separation from parent, bullying, financial difficulties, and being kept off school contain only prospective data. The ACEs of abuse and witnessed abuse contain only retrospective data. The remaining ACEs of neglect, substance abuse by family member, family conflict, and divorce or separation of parents contain a mix of prospective and retrospective data.

**Figure 3.**
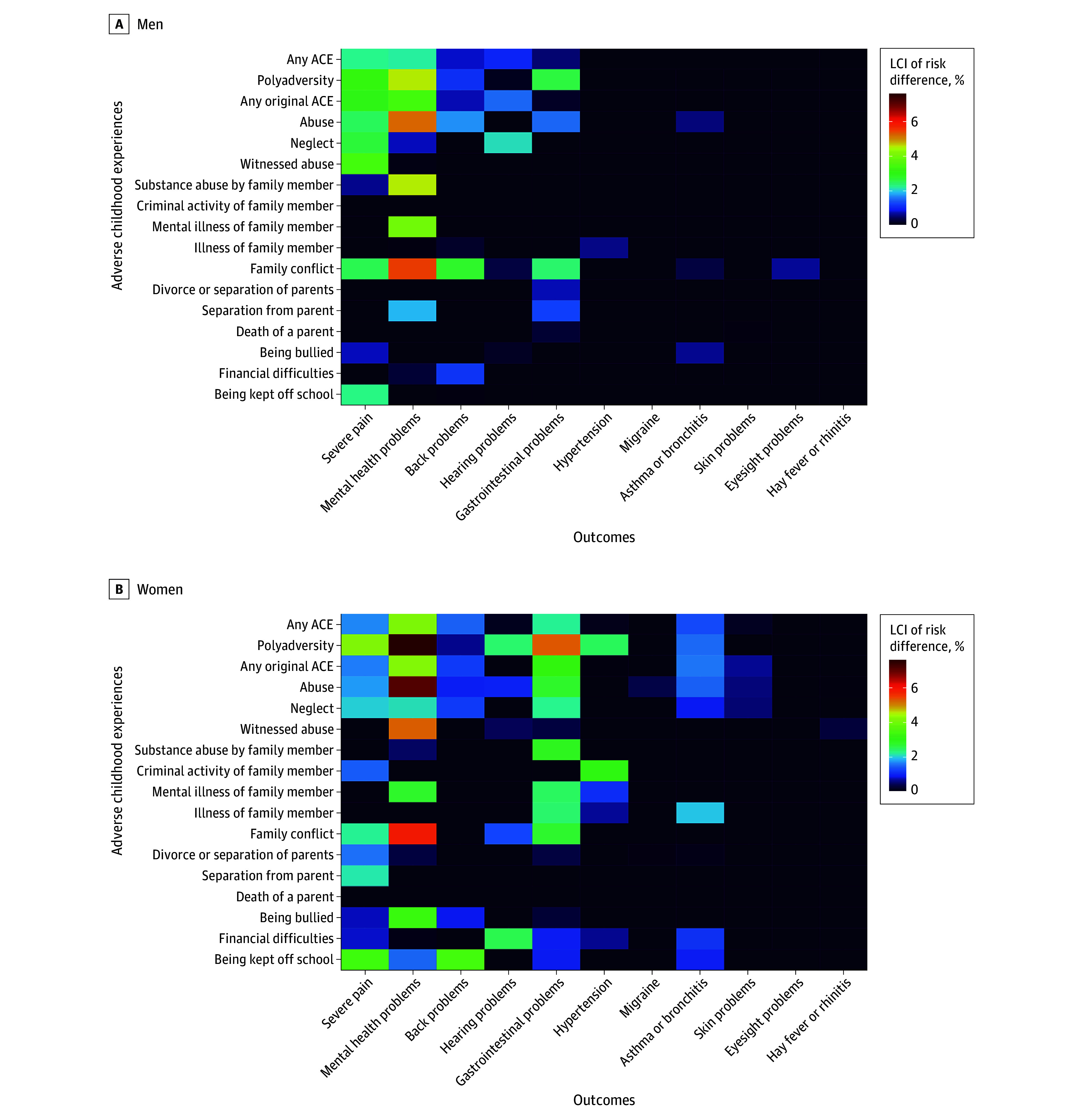
Lower Confidence Bounds of Risk Differences for Individual Health Outcomes, Comparing Pseudopopulations Exposed or Unexposed to Different Categories of Adverse Childhood Experiences (ACEs) Negative values have been set to 0 for ease of visual interpretation. Individual ACEs were determined from a mix of prospective and retrospective data. The ACEs of criminal activity of family member, mental illness of family member, illness of family member, death of a parent, separation from parent, bullying, financial difficulties, and being kept off school contain only prospective data. The ACEs of abuse and witnessed abuse contain only retrospective data. The remaining ACEs of neglect, substance abuse by family member, family conflict, and divorce or separation of parents contain a mix of prospective and retrospective data. LCI indicates lower confidence interval.

Figure 2 shows a heat map of the risk differences for individual ACEs and individual health outcomes, with lower 95% CIs (as a visual representation of the degree of uncertainty) represented in Figure 3. In men, the highest excess risks for poor mental health (with low uncertainty) were associated with reports of abuse, family conflict, substance abuse, or poor mental health in a family member; for severe pain, the largest excess risks (with low uncertainty) were observed for abuse and witnessing abuse, neglect, family conflict, and being kept off school. In women, the highest excess risks for poor mental health (with low uncertainty) were associated with reports of abuse, family conflict, or poor mental health in a family member. The experience of abuse, neglect, family conflict (with low uncertainty), and criminal activity of a family member (with high uncertainty) showed the most wide-ranging associations.

## Discussion

This cohort study using an outcome-wide analytic approach modeled the long-term association of ACEs with health outcomes of a UK birth cohort. In both sexes, severe pain and poor mental health showed the largest excess risks, while in women, gastrointestinal problems and asthma or bronchitis were also associated with ACEs. In contrast, migraine, hay fever or rhinitis, eyesight problems, or skin problems were not associated with ACEs. Polyadversity showed risk increases to a greater extent. Excess risk was similar whether the original set of ACEs was considered or a wider definition used. The experience of abuse, neglect, and family conflict showed the most wide-ranging associations.

This study benefited from the whole-population, birth cohort design of NCDS, which includes a wide range of health outcomes. By undertaking an outcome-wide approach, we were able to evaluate the association of ACEs with multiple health domains within a single analytic framework to contrast magnitudes of associations. Using doubly robust methods and multiple imputation to address confounding and missing data enhanced the validity of our findings.

The findings align with existing evidence showing associations of ACEs (particularly multiple ACEs) with adverse mental and physical health outcomes. Hughes et al^4^ reported associations with all reported health outcomes in their meta-analysis. Our current original research adds to the findings from their review. We have reported on health outcomes that mainly have not been (or commonly) examined by previous studies, such as pain (including back pain and migraine), which is among the leading global causes of years of life lived with disability.^20^ We examined the associations separately by men and women and found that the absolute risk increase associated with multiple ACEs (among conditions associated with ACEs) was always higher in women. Finally, while all 23 outcomes examined by Hughes et al were associated with ACEs, we show that poor mental health and severe pain are outcomes associated with the highest excess risk, while several other health outcomes were not associated with ACEs.

Our analysis of mortality risk showed that the report of an ACE was also associated with having died by the time of follow-up. Despite analyzing survivors at age 50 years, we still observed excess risks in our sample, whereas typically, the anticipated impact of survival bias would be an attenuated effect size. It is possible, though, that any such bias may have exerted an inconsistent influence across outcomes.

Our finding of no apparent increased risk of migraine is at odds with the literature and may reflect differences in study design. For example, the studies in a previous systematic review included only 3 prospective studies, and only a minority of all included studies attempted to adjust for confounding.^21^ It may also reflect the measurement of migraine in the NCDS, which did not distinguish between types or chronicity. Likewise, we were unable to disaggregate the experience of hearing problems reported by NCDS participants or the age of onset. Previous studies have suggested a role of the hypothalamic-pituitary-adrenal axis in chronic tinnitus.^22^ On the other hand, people with childhood-onset hearing problems or deafness may, as a result, be more likely to be exposed to ACEs.^23^

This study partially supports the hypothesis by Von Korff et al^9^ that ACEs may act as broad-spectrum risk factors for multiple health outcomes. Previous authors have pointed to myriad consequent social and behavioral processes that may follow ACEs (eg, lower educational attainment, reduced social support, adoption of health-harming behaviors) as the mechanistic link to the variety of poor health outcomes.^2^ Poor mental health may itself be a mediator on the causal pathway between ACEs and these conditions,^24,25^ but there is also the suggestion that epigenetic, immunologic, or endocrine adaptations or maladaptations may influence the stress response, thereby increasing the risk of chronic widespread pain or gastrointestinal disorders.^26,27,28,29^ To aid interpretation, Figure 2 and Figure 3 suggest that approximately one-third of the cases of poor mental health and severe pain (in the imputed data) would have been prevented had none of the population been exposed to ACEs (as estimated by dividing the difference between the observed and unexposed risks by the observed risk). We were unable to decompose further the associations between the health conditions, given the cross-sectional collection of outcome data, but this would be an important area for future inquiry, particularly to elucidate the role of poor mental health.

This study looked at a wider range of ACEs than typical. For example, we are not aware that the experience of being kept off school to help at home has previously been reported in the context of adversity and was associated with a range of poor health outcomes in women. Interestingly, the excess risk observed was similar irrespective of whether the original or expanded set of ACEs was used, particularly among women, providing support for including a wider range of adverse experiences.

### Limitations

Several methodological issues need to be considered. First, the reliance on retrospective self-report for some ACEs may introduce recall bias, particularly given the long time intervals involved. Two ACEs were derived solely from retrospective reporting (abuse, witnessing abuse), 4 from a mixture of prospective and retrospective data (neglect, substance abuse by a family member, family conflict, divorce or separation of parents), while all others were derived only from prospective data. The results show an association of ACEs with poor health outcomes even for those for which the data collection was entirely prospective. However, the ACEs that showed the largest risk differences and most wide-ranging associations involve at least some retrospective data, echoing previous comparisons in the literature of retrospectively and prospectively gathered ACE data and prompting the suggestion that these data identify nonoverlapping populations.^30^ For this reason, we believe that the inclusion of both forms of data is important, although it complicates interpretation. Second, while the analytic approach accounted for confounders measured at birth, residual confounding cannot be excluded, particularly with regard to socioeconomic and environmental factors. However, the specificity of associations observed argues against the results being explained by residual confounding. Third, longitudinal study designs can suffer biases due to attrition and survival. We used multiply imputed data rather than complete-case analyses and separately analyzed mortality risk. Finally, the study’s restriction to a predominantly White UK population (reflecting the population at the time of initial data collection) limits the generalizability of findings.

## Conclusions

This cohort study provides evidence of the multifaceted potential health consequences of ACEs, with the greatest excess risks associated with poor mental health and severe pain. This finding emphasizes the role of childhood adversity as a key driver of health inequities and a public health priority. The findings underscore the importance of considering prevention strategies to mitigate the potential long-term health consequences of ACEs. Screening for ACEs in primary care settings and targeted interventions for at-risk individuals may help reduce the burden of chronic pain, poor mental health, and other poor health outcomes. If ACEs are indeed broad-spectrum risk factors with multiple causal pathways to multiple health outcomes, a broad-spectrum intervention is warranted. There may also be a need for tailored approaches accounting for sex differences in ACE-associated vulnerabilities, as specific ACEs were shown to have the most wide-ranging associations.

## Notes

Supplement 1. **eTable 1.** Derivation of Adverse Childhood Experience Variables From Items in the National Child Development Study **eMethods.** Imputation Methods for Missing Data **eTable 2.** Characteristics of Complete and Noncomplete Cases **eTable 3.** Variables Included in the Imputation Model **eTable 4.** Factors Associated With Death Prior to Age 50 Years Follow-Up (Multivariable Model) **eTable 5.** Risk and Risk Differences of Health Conditions Associated With Reporting an ACE
eReference.

Supplement 2.
Data Sharing Statement
