# Supplementary material for: Adverse Childhood Experiences and Health at Age 50 Years in the National Child Development Study
Source: JAMA Netw Open. 2025 Aug 28;8(8):e2525708. doi: 10.1001/jamanetworkopen.2025.25708 (PMC12395314; doi:10.1001/jamanetworkopen.2025.25708)
Supplement: Supplement 1. — eTable 1. Derivation of Adverse Childhood Experience Variables From Items in the National Child Development Study eMethods. Imputation Methods for Missing Data eTable 2. Characteristics of Complete and Noncomplete Cases eTable 3. Variables Included in the Imputation Model eTable 4. Factors Associated With Death Prior to Age 50 Years Follow-Up (Multivariable Model) eTable 5. Risk and Risk Differences of Health Conditions Associated With Reporting an ACE eReference. [file jamanetwopen-e2525708-s001.pdf]

Supplemental Online Content

Timmins KA, MacDonald R, Beasley M, Macfarlane GJ. Adverse childhood experiences and health at age 50 years in the National Child Development Study. *JAMA Netw Open*. 2025;8(8):e2525708. doi:10.1001/jamanetworkopen.2025.25708

eTable 1. Derivation of Adverse Childhood Experience Variables From Items in the National Child Development Study

eMethods. Imputation Methods for Missing Data

eTable 2. Characteristics of Complete and Noncomplete Cases

eTable 3. Variables Included in the Imputation Model

eTable 4. Factors Associated With Death Prior to Age 50 Years Follow-Up (Multivariable Model)

eTable 5. Risk and Risk Differences of Health Conditions Associated With Reporting an ACE

This supplemental material has been provided by the authors to give readers additional information about their work.

© 2025 Timmins KA et al. *JAMA Network Open*.

eTable 1. Derivation of Adverse Childhood Experience Variables From Items in the National Child Development Study

ACE	Timing of data collection	Item	Collection method	Age(s)	Presence defined as:
Abuse	Retrospective	Sexually abused by parent	Study member questionnaire	44	1 (‘yes’)
		Physically abused by parent	Study member questionnaire	44	1 (‘yes’)
		Verbally abused by parent	Study member questionnaire	44	1 (‘yes’)
		Suffered humiliation, ridicule, bullying or mental cruelty from parent	Study member questionnaire	44	1 (‘yes’)
Neglect	Combined prospective & retrospective	Child appears scruffy & dirty, underfed	Teacher questionnaire	7, 11	3 = ‘looks underfed’<br>5 = ‘scruffy & dirty’
		Mother/father hardly takes child out	Parent (or parent substitute) interview	11	4 ‘hardly ever’
		Mother/father rarely reads to child	Parent (or parent substitute) interview	11	4 ‘hardly ever’
		Parent shows little interest in child’s education	Teacher questionnaire	7, 11, 16	5 ‘little interest’ OR 4 ‘little interest’
		“I was neglected”	Study member questionnaire	44	1 (‘yes’)
Witnessed Abuse	Retrospective	Witnessed physical or sexual abuse of others in my family	Study member questionnaire	44	1 (‘yes’)
Substance Abuse by Family Member	Combined prospective & retrospective	Family difficulties: alcoholism	Parent (or parent substitute) interview	7	2 (‘yes’)
		Mother had trouble with drinking or drug use	Study member questionnaire	44	1 (‘yes’)
		Father had trouble with drinking or drug use	Study member questionnaire	44	1 (‘yes’)
Criminal Activity of Family Member	Prospective	Family has had contact with probation officer	Parent (or parent substitute) interview	7	2 (‘yes’)
Mental illness of Family Member	Prospective	Family experienced mental illness difficulties	Parent (or parent substitute) interview	7	2 (‘yes’)
		Family experienced difficulties with ‘mental subnormalities’	Parent (or parent substitute) interview	7	2 (‘yes’)

© 2025 Timmins KA et al. *JAMA Network Open*.

ACE	Timing of data collection	Item	Collection method	Age(s)	Presence defined as:
		Family has had contact with psychiatric social worker	Parent (or parent substitute) interview	7	2 (‘yes’)
		Family has had contact with mental welfare officer	Parent (or parent substitute) interview	7, 11	2 (‘yes’)
Illness of Family Member	Prospective	Family experienced illness/disability difficulties	Parent (or parent substitute) interview	7	2 (‘yes’)
		Age category at onset of mother’s chronic illness	Parent (or parent substitute) interview	11	≥1
		Age category at onset of father’s chronic illness	Parent (or parent substitute) interview	11	≥1
		Number weeks off work for father through illness	Parent (or parent substitute) interview	11, 16	≥4
		Chronic illness in house since child	Parent (or parent substitute) interview	16	3 ‘yes but not now’<br>4 ‘still continuing’
Family Conflict	Combined prospective & retrospective	Family difficulties: domestic tension	Parent (or parent substitute) interview	7	2 (‘yes’)
		There was much conflict & tension in household when growing up	Study member questionnaire	44	1 ‘a lot’
Divorce or Separation of Parents	Combined prospective & retrospective	Divorce/separation	Parent (or parent substitute) interview	7	2 (‘yes’)
		Divorce/separation	Study member interview	33	2 (‘yes’)
		Why child away from mother/father	Parent (or parent substitute) interview	11, 16	10 ‘broken marriage’<br>OR<br>2 ‘divorced’<br>3 ‘separated’
Death of a Parent	Prospective	Death (why child away from own/adoptive mother/father)	Parent (or parent substitute) interview	11, 16	11 (‘death’)<br><br>OR 1 (‘death’)
		Family difficulties: death of father/mother	Parent (or parent substitute) interview	7	2 (‘yes’)
		Yr between onset of illness and mother/father death (categories)	Parent (or parent substitute) interview	7	1-5

© 2025 Timmins KA et al. *JAMA Network Open*.

ACE	Timing of data collection	Item	Collection method	Age(s)	Presence defined as:
Separation from Parent	Prospective	Why child away from mother/father	Parent (or parent substitute) interview	11, 16	9 ‘illegitimacy’<br>12 ‘other’<br>OR<br>4 ‘illegitimate’<br>5 ‘other’<br>6 ‘not home, in care’
		Child ‘in care’	Parent (or parent substitute) interview	7	2 ‘now LA care’<br>3 ‘past LA care’<br>5 ‘now voluntary care’<br>6 ‘past voluntary care’<br>7 ‘in care abroad’
		Ever been in LA care	Parent (or parent substitute) interview	11, 16	1 ‘yes in care now’<br>2 ‘yes only in past’
		Ever been in voluntary services care	Parent (or parent substitute) interview	11, 16	1 ‘yes in care now’<br>2 ‘yes only in past’
Bullying	Prospective	How often child bullied by other kids	Parent (or parent substitute) interview	7	2 ‘frequently’
		Is child bullied by other children	Parent (or parent substitute) interview	11	3 ‘frequently’
Financial Difficulties	Prospective	Family experienced financial difficulties	Parent (or parent substitute) interview	7	1 ‘yes’
		Serious financial hardship in past year	Parent (or parent substitute) interview	11, 16	1 ‘yes’
Being Kept Off School	Prospective	Kept off school to help at home	Parent (or parent substitute) interview	16	1 ‘yes’

© 2025 Timmins KA et al. *JAMA Network Open*.

eMethods. Imputation Methods for Missing Data

We followed the guidance of Silverwood et al.[1] eTable2 presents characteristics of complete versus non-complete participants. Participants with complete data included more females, more people whose mother had stayed at school beyond the minimum school leaving age, fewer people whose mother had smoked during pregnancy, and fewer people reporting a mental health problem at age 50.

Assuming data were Missing At Random (MAR), we used multiple imputation with chained equations to impute missing values for 20 imputations. Imputation models were stratified by sex, using the by() option. We used a default burn-in of 10 iterations. Participants who had died prior to age 50 were excluded from the sample prior to imputation. Variables included in the imputation model are shown in eTable 2. With the exception of sex, these variables were all imputed. Auxiliary variables were identified from the suggestions of Silverwood et al that were found to be predictors of missingness in the data set; we selected those which were also predictors of missingness of chronic pain. To minimise collinearity, we narrowed this down to 7 auxiliary variables. Model performance was inspected by comparing observed and imputed values, checking trace plots, and examining post-estimation variances.

eTable 2. Characteristics of Complete and Noncomplete Cases

	Participants with complete[a] data	Participants with missing data
% female	2636 (53%)	5354 (47%)
White ethnicity	4906 (99%)	11219 (99%)
Birthweight	118 (18)	117 (19)
Mother smoked during pregnancy	1543 (31%)	3678 (34%)
Mother's age at birth	27.7 (5.5)	27.3 (5.8)
Mother stayed at school beyond minimum school leaving age	1381 (28%)	2669 (24%)
Crowding (people per room, mean)	1.4 (0.9)	1.6 (1.0)
Severe pain	376 (8%)	322 (9%)
Mental health problem	599 (12%)	644 (14%)
Asthma	467 (9%)	458 (10%)

[a] With data complete for the following variables : mother/father had trouble with drinking or drug use; conflict & tension while growing up; being neglected; verbally abused by parent; suffered humiliation, ridicule, bullying or mental cruelty from parent; witness physical or sexual abuse of other in the family; physically abused by parent; sexually abused by parent; suffered another type of mistreatment; child appeared scruffy & dirty or underfed; mother/father hardly takes child out; mother/father rarely reads to child; parent shows little interest in child's education; family difficulties with alcoholism; family contact with probation officer/mental welfare officer; family experienced mental illness difficulties/ 'mental abnormalities'/psychiatric social worker; family experienced difficulties with illness/disability; mother/father had chronic illness; father was off work for ≥4 weeks with illness; chronic illness in house; family difficulties with domestic tension; divorce/separation of parents; child was separated from mother/father; death of mother/father; years between onset of illness and mother/father death; been in (local authority/voluntary services) care; frequency child bullied by other children; family experienced financial difficulties; family had serious financial hardship in past year; child kept off school to help at home.

© 2025 Timmins KA et al. *JAMA Network Open*.

eTable 3. Variables Included in the Imputation Model

Variable	Role	Imputation method	% missing
Abuse	Exposure	Logit	46
Neglect	Exposure	Logit	3
Witnessed domestic abuse	Exposure	Logit	46
Substance abuse by family member	Exposure	Logit	12
Criminal activity by family member	Exposure	Logit	15
Mental illness of a family member	Exposure	Logit	5
Illness of family member	Exposure	Logit	4
Family conflict	Exposure	Logit	7
Divorce/separation of parents	Exposure	Logit	3
Death of a parent	Exposure	Logit	4
Separation from a parent	Exposure	Logit	4
Being bullied	Exposure	Logit	8
Financial difficulties	Exposure	Logit	9
Being kept off school	Exposure	Logit	34
Severe pain	Outcome	Logit	48
Pain interference	Outcome	Logit	48
Mental health problems	Outcome	Logit	42
Asthma/bronchitis	Outcome	Logit	42
Hayfever/Rhinitis	Outcome	Logit	42
Back problems	Outcome	Logit	42
Hearing problems	Outcome	Logit	42
Eyesight problems	Outcome	Logit	42
Hypertension	Outcome	Logit	42
Migraine	Outcome	Logit	42
Skin problems	Outcome	Logit	42
Gastrointestinal problems	Outcome	Logit	42
Sex	Moderator	By (stratification)	0
Birthweight	Confounder	Regress	6
Mother's age at birth	Confounder	Regress	2
Mother's smoking during pregnancy	Confounder	Mlogit	3
Mother's schooling beyond minimum age	Confounder	Logit	2
Crowding in the home	Confounder	Regress	5
Mother worked when study member aged <5 years	Auxiliary	Mlogit	20
Cognitive ability summary at age 7	Auxiliary	Regress	18
Number of people <21yrs in household at age 7	Auxiliary	Regress	19
School attendance at age 16	Auxiliary	Regress	33
Hospital admissions at age 33	Auxiliary	Regress	38
Access to a car/van at age 42	Auxiliary	Mlogit	38
Number of children living with at age 46	Auxiliary	Regress	51

© 2025 Timmins KA et al. *JAMA Network Open*.

eTable 4. Factors Associated With Death Prior to Age 50 Years Follow-Up (Multivariable Model)

Variable	Units	Mortality Risk Ratio	95% Confidence Interval
Any Adverse Childhood Experience	No	-	-
	Yes	1·22	1·01 – 1·47
Sex	Male	-	-
	Female	0·58	0·49 – 0·69
Birthweight	Ounces	0·995	0·99 – 0·9998
Mother's Age at childbirth	Years	0·98	0·97 – 0·996
Mother Smoking	Never Smoked	-	-
	Prior to Pregnancy	1·53	1·15 – 2·04
	During Pregnancy	1·25	1·05 – 1·48
Mother at School Beyond Minimum Age	No	-	-
	Yes	0·95	0·78 – 1·16
Overcrowding: People per room in household at time of birth	Range: 1 – 6	1·13	1·06 – 1·21

© 2025 Timmins KA et al. *JAMA Network Open*.

eTable 5. Risk and Risk Differences of Health Conditions Associated With Reporting an ACE

Health Outcome	Male				Female			
	Observed[a] risk of outcome	Risk unexposed population	Risk exposed population	Risk Difference 95% CI	Observed[a] risk of outcome	Risk unexposed population	Risk exposed population	Risk Difference 95% CI
Severe pain	7·76	4·88	8·70	3·82 (2·23, 5·42)	10·28	7·53	11·22	3·69 (1·71, 5·67)
Mental ill-health	9·63	6·68	10·53	3·85 (2·16, 5·55)	17·51	12·59	19·10	6·50 (4·13, 8·88)
Asthma/bronchitis	8·76	8·36	8·93	0·57 (-1·21,2·35)	11·67	8·21	12·60	3·39 (1·30, 5·49)
Hayfever/rhinitis	13·21	15·17	12·56	-2·61 (-4·84, 0·39)	13·88	13·74	13·93	0·18 (-2·29, 2·65)
Back	18·22	15·80	19·11	3·32 (0·78, 5·85)	18·06	15·12	18·97	3·85 (1·48, 6·21)
Hearing	12·36	10·20	13·07	2·87 (1·02, 4·72)	7·80	6·43	8·35	1·92 (0·15, 3·68)
Eyesight	65·06	65·90	64·82	-1·28 (-4·22, 1·67)	68·39	68·09	68·25	0·16 (-3·05, 3·37)
Hypertension	17·14	15·82	17·65	2·03 (-0·57, 4·63)	14·06	12·20	14·52	2·32 (0·12, 4·51)
Migraine	5·23	4·31	5·48	1·17 (-0·19, 2·53)	12·07	11·52	12·20	0·68 (-1·42, 2·78)
Skin	7·87	7·65	7·88	0·23 (-1·45, 1·91)	8·97	7·57	9·43	1·87 (0·18, 3·56)
Gastrointestinal	7·58	5·96	8·13	2·17 (0·53, 3·82)	9·57	6·64	10·63	3·99 (2·19, 5·79)

CI: Confidence Interval. ACE: Adverse Childhood Experience.

[a] % of individuals in the imputed data who have the outcome

© 2025 Timmins KA et al. *JAMA Network Open*.

References

Silverwood R, Narayanan M, Dodgeon B, Ploubidis G. Handling missing data in the national child development study: user guide (version 2). London: UCL Centre for Longitudinal Studies. 2021.

© 2025 Timmins KA et al. *JAMA Network Open*.
