# Supplementary material for: Adverse Childhood Experiences and Health at Age 50 Years in the National Child Development Study
Source: JAMA Netw Open. 2025 Aug 28;8(8):e2525708. doi: 10.1001/jamanetworkopen.2025.25708 (PMC12395314; doi:10.1001/jamanetworkopen.2025.25708)
Supplement: Supplement 2. — Data Sharing Statement [file jamanetwopen-e2525708-s002.pdf]

Data Sharing Statement

Data
Data available: No

Additional Information
Explanation for why data not available: Most data from the NCDS are freely available through the UK Data Service (https://ukdataservice.ac.uk). A general analysis plan covering all the cohorts analyzed as part of CAPE is available on the OSF: https://www.osf.io/shdy3. Stata code for the analyses in the final article will be shared on the OSF projects site. The data used are from the National Childhood Development Study, and researchers are able to make application to NCDS for access to data.
